# Assessing the Feasibility of Biorefineries for a Sustainable Citrus Waste Management in Korea

**DOI:** 10.3390/molecules29071589

**Published:** 2024-04-02

**Authors:** Sang-Hwan Lee, Seong Hee Park, Hyun Park

**Affiliations:** 1 Technical Research Institute, Jeju BioRefine, Jeju 63148, Republic of Korea; soillsf@gmail.com; 2 Technical Research Institute, Fine Korea Corp., Seoul 07294, Republic of Korea; indipark62@hanmail.net; 3 Department of Biotechnology, College of Life Sciences and Biotechnology, Korea University, Seoul 02841, Republic of Korea

**Keywords:** biorefinery, *Citrus reticulata*, environmental pollution, mandarin, waste valorization

## Abstract

Citrus fruits are one of the most widely used fruits around the world and are used as raw fruits, but are also processed into products such as beverages, and large amounts of by-products and waste are generated in this process. Globally, disposal of citrus waste (CW) through simple landfilling or ocean dumping can result in soil and groundwater contamination, which can negatively impact ecosystem health. The case of Korea is not much different in that these wastes are simply buried or recycled wastes are used as livestock feed additives. However, there are many reports that CW, which is a waste, has high potential to produce a variety of products that can minimize environmental load and increase added value through appropriate waste management. In this study, we aim to explore the latest developments in the evaluation and valorization of the growing CW green technologies in an effort to efficiently and environmentally transform these CW for resource recovery, sustainability, and economic benefits. Recent research strategies on integrated biorefinery approaches have confirmed that CW can be converted into various bioproducts such as enzymes, biofuels and biopolymers, further contributing to energy security. It was found that more efforts are needed to scale up green recovery technologies and achieve diverse product profiling to achieve zero waste levels and industrial viability.

## 1. Introduction

The emerging “bioeconomy” is a concept coined by the European Commission in 2012 to address the use of renewable biological resources as economically viable products and bioenergy, as described by Ravindran and Jaiswal [1]. Biorefining is an integrated, efficient, and flexible approach that combines biomass conversion processes to produce high-value-added chemicals, fuel, and power from waste biomass.

Biorefineries considerably differ from petroleum refineries, where fossil fuels are converted into value-added products and energy [2]. Many researchers have proposed biorefinery concepts to solve the problem of agricultural (e.g., citrus) waste disposal [3]. Citrus fruits offer nutritional value and health benefits to consumers because they contain large amounts of secondary metabolites [4]. Additionally, citrus fruits (genus *Citrus* L. of the Rutaceae family) are among the most abundantly grown and consumed fruits worldwide. The citrus family includes several fruits, mainly *Citrus sinensis* (orange), *Citrus reticulata* (mandarin), *Citrus tangerine* (tangerine), *Citrus aurantifulia* (lime), *Citrus limon* (lemon), *Citrus limetta* (sweet lime), and *Citrus paradisi* (grapefruit) [5,6,7]. In 2016, more than 124 million tons of citrus were produced worldwide, of which 50–60% were consumed as fresh fruit; the remaining 40–50% were used for industrial processing [8,9].

Citrus processing by-products, generated in large quantities, are unique to citrus waste (CW). Improper disposal of this waste through conventional methods (e.g., incineration and landfill) creates substantial environmental and economic burdens [10,11,12]. However, environmentally friendly waste management is expensive and considerably increases the annual budgets of municipalities and the agricultural industry.

Given the negative impact of CW production on the environment and the contributions of CW-derived products to the bioeconomy, valorization of CW is essential [8]. Global research efforts have attempted to reduce the environmental burden of CW treatment and add new value; however, thus far, the focus has been mainly on sweet orange. Few CW studies have focused on mandarin fruit.

Over the years, researchers have attempted to develop various processing methods for complete exploitation of CW [8,9,13]. However, these efforts have met resistance because greater awareness is needed to change conventional attitudes towards practices with bioeconomic merit. However, if the potential of CW valorization by green economy schemes is realized, the negative impacts of citrus processing on the environment may be lessened. Consistent with this global trend, research and development related to CW biorefining has recently begun in Korea.

The main purpose of this study was to investigate the feasibility of introducing a biorefinery system for the proper management and utilization of CW, focusing on mandarin, which is mainly cultivated in Korea. We also investigated the constraints and improvements needed for mandarin waste biorefinery systems to provide other stakeholders with insights into the most suitable solutions for the economic and environmental sustainability of citrus residue management.

## 2. Environmental Issues Related to Citrus Production and Its By-Products in Korea

In Korea, citrus are produced only in Jeju. The main cultivated variety is mandarin, *Citrus reticulata*. Mandarin is a fruit that has been grown in east Asia for thousands of years; it contains abundant nutrients and bioactive substances with potential medicinal value [14,15]. In the mandarin family, along with common mandarins, there are various types and hybrids created mostly through natural mutations [16]. Recently, the use of hard-to-peel citrus fruits such as orange, grapefruit, and lime has decreased, whereas the production and consumption of easy-to-peel mandarin-type citrus fruits are increasing [17]. Global mandarin production and consumption have increased, according to the United States Department of Agriculture 2020 Citrus Trade and Marketing Report over the past decade, global mandarin production has increased from 22 million tons in 2010 to 31.6 million tons in 2020; consumption has increased from 20 million tons to 30 million tons during the same period [18].

Mandarin fruit is a rich source of phytochemicals, including both primary and secondary metabolites [19]. Its primary metabolites are mainly composed of sugars, organic acids, lipids, and vitamins as macronutrients; its secondary metabolite classes include phenolics, flavonoids, and limonoids that contribute to its flavor and health benefits (Table 1) [20,21].

Recent data show that the mean production levels of fresh mandarin fruit and mandarin waste per year are approximately 600,000 and 60,000 tons, respectively, in Korea (Table 2).

**Figure 1:**
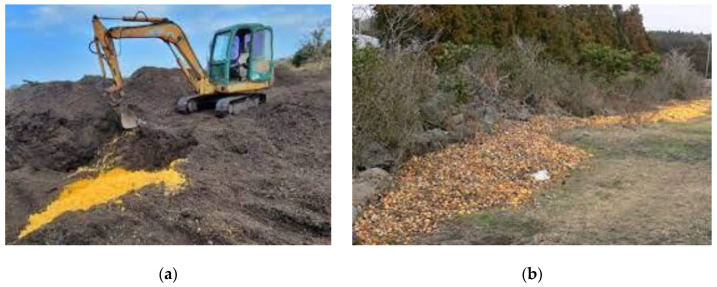
Citrus waste (CW) generation in Korea: (**a**) landfill disposal of production waste and (**b**) local disposal of discarded waste.

In Korea, CW is generated in two ways: as peel and pulp after processing and as waste generated during local disposal due to damage or poor quality (Figure 1). According to the National Agricultural Cooperative Federation of Korea, the amount of discarded citrus in production areas was 40,168 tons in 2021.

Mandarin consumption for juice production results in peel waste that constitutes approximately 30% of the mass of mandarin fruit [22]. Unfortunately, most mandarin peel waste is currently dumped in landfills or marine environments, creating serious environmental problems and incurring substantial disposal costs [23].

CW has a low pH (3–4), high organic composition (95% of total solids), and high moisture content (~80–90%) [8,24]. CW includes fats, free sugars (e.g., glucose, fructose, and sucrose), organic acids, carbohydrate polymers (cellulose, hemicellulose, and pectin), enzymes (pectinesterase, phosphatase, and peroxidase), flavonoids, sugar, essential oils (mainly limonene), and pigments [25]. CW contains approximately 1% of the dry weight of organic acids (e.g., citric, malic, malonic, and oxalic acids) [26,27]. Since CW has a low pH and high organic content, methane is generated under anaerobic conditions; methane has a global warming potential 25-fold greater than CO_2_ [28]. In addition to solid waste, the wastewater generated during CW processing has a negative impact on the environment due to its high biological and chemical oxygen demand; citrus wastewater also contains phenolic compounds and various organic acids [29]. Solid CW generated during the production and processing of citrus fruits is generally disposed of on-site or landfilled, and liquid CW generated during processing is currently generally disposed of through environmentally hazardous sewage. Due to the increasing amount and unique characteristics of CW, existing waste treatment methods face environmental and economic challenges [10,12]. Conventional CW management methods (incineration and landfill) are insufficient and problematic in terms of mitigating the environmental impact of CW and maintaining energy efficiency [8,11]. The waste generated from agriculture-based industries, including major industries related to citrus fruit processing, strongly impacts socioeconomic development. However, views toward this waste vary among industries. For example, although it uses a substantial amount of water and produces a large amount of waste, the winemaking industry has developed a positive public perception due to its socioeconomic and cultural benefits.

To minimize management costs and prevent environmental damage, numerous studies have been conducted regarding the viability of agricultural waste recycling through by-products. In recent decades, there has been considerable interest in CW valorization; however, progress toward this goal has been slow [12]. An important aspect required to develop a reliable waste management plan is comprehensive knowledge of the nature and quantity of the waste to be managed.

## 3. Current Status of Citrus Waste Biorefining Efforts

The by-products generated from citrus, a rich source of bioactive compounds, include essential oils, pectin, and water-soluble and -insoluble antioxidants. The citrus fruit consists of 87% water and 13% other constituents composed of fats, essential oils, minerals, glucosides, fibers, pentosans, citric acid, and pectin [9,30,31]. CW contains large amounts of valuable bioactive substances, including high-quality fiber, pectin, and bioactive compounds (e.g., flavonoids, phenolic acids, carotenoids, and essential oils) [4,16,32,33,34,35,36]. In the biorefining process, CW can be converted into high-value chemicals, fuels, food additives, and polymer precursors through chemical and biological approaches, which can be utilized in various industries such as food, pharmaceuticals, and cosmetics.

**Figure 2:**
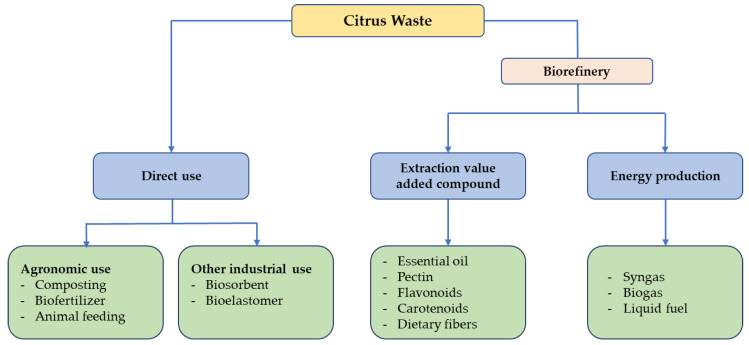
Schematic of CW biorefining.

CW valorization is achieved through three main platforms: direct use of basic/modified CW, biochemical processes, and green extraction technologies (Figure 2). Regarding the current CW biorefining status, here we consider research and development trends, focusing on the direct use (i.e., minimal modification) and production of high-value-added materials, biogas, and bioethanol.

### 3.1. Direct Use

#### 3.1.1. Agronomic Utilization

Direct agricultural uses of CW include using it as livestock feed, spraying it directly on farmland, or processing it after composting.

The most widely used agricultural application of CW is as animal feed, where CW (wet, ensiling or dry) is used as a partial replacement or additive to conventional animal feed, such as grains. It has been reported that CW can increase the digestibility of food due to its high pectin content and low lignin content, and that when fed to pigs, etc., meat quality is improved without affecting animal growth [37,38].

Agricultural use of CW by spraying it directly on the land can be said to be an appropriate method to increase the organic matter content of the soil and consequently improve fertility. Addition of organic matter such as CW improves soil resistance to raindrop impact and water infiltration capacity, thereby reducing water runoff and soil erosion. The presence of organic matter also leads to higher water holding capacity, greater porosity, air permeability, and improved nutrient utilization efficiency of the soil. However, the problem is that the antibacterial activity of EO and other bioactive molecules contained in CW can be very harmful to soil microorganisms. Composting can be said to be a way to overcome the problems of direct spraying. From the perspective of composting as an environmentally friendly CW treatment method, composting of organic residues generated from CW has recently been shown to have a positive impact on agriculture. This was found in research [39,40]. Similar to other vegetable matrices, CW can be utilized to produce good quality compost. However, composting conditions must be adjusted considering the characteristics of CW. Regarding CW composting, Lòpez et al. [26] recommended that the carbon/nitrogen (C/N) ratio, pH, and moisture content of the waste be adjusted to 24:1, 6.3, and 60%, respectively. Golueke [41] suggested that pH and C/N ranges of 6.0–7.5 and 25–35 are appropriate.

#### 3.1.2. Other Industrial Uses

Research has been conducted to utilize CW, especially peels, as activated carbon and biochar. Citrus peels can be used as an inexpensive biosorbent for treatment of contaminated water; thus, they represent an environmentally friendly solution for CW management [42,43,44]. The development of nanomaterials from food processing waste is a very new field of research; thus far, there have been few studies concerning the use of CW for nanomaterial preparation. Balu et al. [45] used microwave treatment to extract several compounds, obtaining mainly mesoporous cellulose with large macropores. In another study, Mariño et al. [46] extracted nanocellulose with a mean diameter of 10 nm and length of 458 nm from citrus fruit waste. Hiasa et al. [47] obtained cellulose nanofibrils with a width of 2–3 nm using purified cellulose from citrus peel waste. Zhang and Zhou [48] created microspheres from pectin-derived nanofibrils. Fan et al. [49] used CW as a substrate to produce nanobacterial cellulose. Lee et al. [50] manufactured a functional bioelastomer with antioxidant and antibacterial activity from citrus peels; they confirmed that this material was effective in terms of maintaining mechanical strength when added to the bioelastomer.

### 3.2. Value-Added Compound Production

Applications that use the whole citrus peel without distinguishing individual components (such as those involving animal feed, organic fertilizer, or compost base) represent the most common and simplest way to process raw citrus material [26]. Multiple commercially important, high-value-added compounds extracted from CW are utilized in the food, pharmaceutical, and cosmetic industries [51]. Representative compounds include essential oils, pectin, and flavonoids. More recently, conversion processes in biorefineries have been proposed for the most profitable and environmentally sound use of CW, leading to the production of various products, as described below.

#### 3.2.1. Essential Oils

Essential oils are mainly distributed in oil sacs or glands with a diameter from 0.4 to 0.6 mm located at irregular depths of the flavedo, corresponding to the outer peel of citrus fruits, and consist of a mixture of terpene hydrocarbons and oxygenated derivatives such as aldehydes, alcohols, and esters [52,53,54].

In general, the recovery rate of the produced oil varies according to extraction method, extraction time, temperature, pressure, and type and quality of plant material; the produced oil contains approximately 0.05 to 5% of essential oil [6]. The essential oil components can vary depending on the cultivar, ripening stage, extraction method applied, and plant part used [55]. According to Feng et al. [56], mandarin contains approximately 35 to 158 mg/L of essential oils. Although small amounts are distributed in other parts of the fruit, such as the juice cells themselves, the oil in the juice cells has better quality than oil in the fruit skin [57].

Citrus peels mainly contain monoterpene hydrocarbons at a level of 86% of the total oil, with a lower content relative to sweet orange (83.9–95.9%), bitter orange (89.7–94.7%), and grapefruit (84.8–95.4%). The most important essential oil in mandarin is limonene (95%), followed by γ-terpinene, α-pinene, β-pinene, β-myrcene, and linalool [58].

These essential oil components of mandarin have a wide range of biological properties, including anti-proliferation and radical scavenging effects, mainly due to their high limonene content. They also exhibit broad antimicrobial properties that inhibit the growth of bacteria such as *Escherichia coli*, *Bacillus subtilis*, *Pseudomonas aeruginosa*, and *Staphylococcus aureus*, as well as fungi including *Penicillium italicum*, *P. digitatum*, *P. chrysogenum*, *Aspergillus niger*, *A. flavus*, *Alternaria alternata*, *Rhizoctonia solani, Curvularia lunata*, *Fusarium oxysporum*, and *Helminthosporium oryzae* [58]. Citrus essential oils and their main component, D-limonene, are used in the cosmetic, pharmaceutical, and chemical industries. For example, citrus essential oils are used to reduce unpleasant tastes in pharmaceuticals, as an aromatic ingredient in toilet soaps, detergents, creams, lotions, perfumes, and other home care products [54,59,60], and as an environmentally friendly solvent [61,62]. Citrus essential oils are also utilized as alternatives to synthetic disinfectants and antibacterial agents; their strong antibacterial activity destroys the cellular integrity of microbial cells and inhibits respiration by those cells [63].

#### 3.2.2. Pectin

In CW, pectin naturally exists in a complex, insoluble form (protopectin). Most pectin in CW is present in the juice bag material, and a substantial amount is distributed in the albedo of the peel. Pectin, also known as pectic polysaccharide, is a heteropolysaccharide block copolymer substituted with a 300–1000 saccharide unit backbone of (1,4-α-linked) galacturonic acid and (1,2-linked) rhamco [64,65].

Pectin, a complex carbohydrate, comprises an α-galacturonic acid polymer with varying numbers of methyl ester groups [66]. It is abundantly distributed in the primary cell walls of most vegetable and fruit plants, and it is water-soluble; thus, it can be used to add texture to foods and beverages [67]. Under low-moisture conditions, pectin can function as a hydrocolloid, trapping water to form a gel. Due to its stabilizing and thickening properties, pectin is also used in foods such as jam; it is considered a suitable matrix for making edible active films, which are widely applied in food packaging due to their biodegradability, biocompatibility, edibility, and chemical diversity. Additionally, pectin as a dietary product reduces lipid absorption [68,69].

Citrus pectin is used to enhance the texture of foods, beverages, medicines, and cosmetics; it is also used as a thickener, emulsifier, and stabilizer in the food industry. Notably, 75% of pectin is used in the manufacture of jams, jellies, marmalade, and similar products [8], and it is widely used as a functional food ingredient (i.e., code E440 in products from the European Union). The pectin industry is worth at least EUR 400 million, with 45 million kg consumed annually worldwide. Finally, pectin is used as a stabilizer for acidified milk and yogurt and as a pharmaceutical detoxifier with anti-diarrheal effects; it also assumes various roles in cosmetics involving gels and pastes [69].

#### 3.2.3. Flavonoids

Flavonoids, which are phenolic compounds naturally produced in plants, are secondary metabolites and there are numerous reports showing that they exhibit antioxidant, anti-inflammatory, anti-mutagenic, anti-aging, and anti-cancer properties, leading to increasing interest in flavonoids [70]. The flavonoid level of mandarin juice is approximately 360 mg/L, slightly higher than the level of lemon juice (320 mg/L) and lower than the levels of orange and grapefruit juices (620 and 550 mg/L, respectively) [71]. Phenolic compounds are present in large quantities in CW. The most important flavonoids are naringin, neoeriocitrin, and hesperidin; other flavonoids include diosmin, rutin, naringenin, eriodictyol, hesperetin, apigenin, luteolin, diosmetin, kaerapferon, quercetin, and tangeretin [51,72]. Hesperidin is the most abundant flavonoid in mandarin juice, comprising 53–75% of the total pulp content; it has excellent analgesic, anti-inflammatory, anti-hypercholesterolemic, anti-hypertensive, and anti-cancer effects. Additionally, hesperidin is used in dyes and cosmetics [19,73,74].

### 3.3. Energy Production

CW is a potential alternative biomass with high pectin content and low lignin content, which can easily be converted into bio-based high-value products without extensive pretreatment for lignocellulosic biomass [75,76]. The low lignin content of CW allows it to be used with conventional biorefinery strategies or in efficient biorefining approaches with advanced integrated management.

Due to the low lignin content and low pretreatment burden, considerable research has recently been conducted regarding the production of biofuels (e.g., ethanol) from CW. Pectin, cellulose, and hemicellulose polysaccharides are hydrolyzed into simple sugars and fermented to produce alcohol [77].

Bioethanol is a biofuel alternative to petroleum [78]. Over the past few years, citrus by-products have been extensively studied for bioethanol production. The production process mainly includes pretreatment steps (fermentation) that involve the efficient release of polysaccharides, hydrolysis/saccharification (i.e., conversion of polysaccharides to monosaccharides), and (finally) fermentation in which sugars are converted into ethanol [57]. However, because the essential oil in CW serves as a major factor hindering bioethanol production, a pretreatment process is likely necessary to remove the oil. For example, a biomass popping pretreatment was developed to efficiently produce bioethanol from *C. unshiu* (Satsuma mandarin) bark using *Saccharomyces cerevisiae*; this treatment successfully reduced the limonene concentration to <0.01% for 46.2 g [78]. In summary, the ethanol production process is a process of pretreating and hydrolyzing pectin, cellulose, and hemicellulose from CW to make monosaccharides, then converting the sugars into ethanol via *Saccharomyces* [79].

Anaerobic digestion of citrus by-products is attracting attention as a promising and sustainable option for biogas production under anaerobic conditions, due to the high methane potential of these residues. Additionally, citrus by-products are the end products of anaerobic digestion, producing nutrient-rich digestates that can be used as soil amendments [80].

Biogas, typically a mixture of methane (50–70%) and carbon dioxide (30–50%), is the main product of anaerobic digestion of organic substrates. Agro-industrial wastes are suitable substrates for biogas production and often contain mineral ions that enhance methane production [81]. The increased biogas production from organic wastes and residues suggests the future utilization potential of CW, whose methane potential is much higher than the potentials of other crop residues [82,83,84]. Panwar et al. [39] produced biogas with yields of up to 322.63 mL biogas/g volatile solids through an integrated process involving treatment of limonene.

## 4. Challenges and Technical Limitations for Biorefinery Implementation

CW can be used directly or through simplified processing as animal feed or soil conditioner. It can also be biorefined, although there are economic constraints because the cost of processing by-products places a substantial financial burden on the citrus processing industry. Implementation requires extensive consideration of the impacts to water resources and the soil environment [85].

Appropriate CW management and the introduction of a biorefinery system are important for sustainability efforts. However, various technical limitations and economic constraints must be addressed for full-scale introduction of CW biorefining.

Composting and production of animal feed, usually without extensive pretreatment, are conventional approaches to solving the environmental problems associated with CW disposal in agriculture. However, CW contains phenolic compounds and limonene that interfere with the activities of aerobic microorganisms. Therefore, untreated CW is unsuitable for composting. Additionally, the phytic acid contained in citrus peel waste functions as a nutritional barrier, limiting its use as animal feed [29,86,87]. Therefore, new processes to remove or convert citrus peel waste are urgently needed to reduce its environmental burden.

For agricultural uses of CW, direct application to land can also be considered, but the antibacterial activities of essential oils and other bioactive substances abundant in CW may have a negative impact on soil microorganisms [85,88,89,90]. Sharma et al. [9] reported that the essential oils present in CW are toxic to yeast and inhibit the growth of beneficial bacteria, yeast, and mold (e.g., *B. subtilis*, *S. cerevisiae*, *A. awamorii*, etc.). Furthermore, if partially composted CW organic matter is spread on the soil, the oxygen in the soil may become depleted, causing a bad odor. Leachate from CW may also contaminate the groundwater supply. Therefore, suitable application protocols, composting, and proper disposal of bioactive compounds are prerequisites for agricultural utilization of CW.

Conventional CW management practices exhibit poor sustainability in terms of cost, valorization, and the extraction of citrus peel-derived bioactive molecules [2,8]. Regarding the CW value-added components, biorefining requires large volumes of fuel to satisfy the total energy demand. Rising energy prices and the need to reduce CO_2_ emissions from fossil fuels require the development of new extraction technologies that are more energy-efficient and solvent-free, thus reducing energy input and eliminating the risk of environmental problems.

The establishment of industrial-scale biorefinery systems is not feasible in the absence of a market, which is a major limiting factor for CW-based biorefining [28]. Although pilot laboratory-scale trials are underway to explore various microbial and biotechnologies for the sustainable and environmentally friendly procurement and production of bioactive molecules in CW biorefineries, their industrial-scale applicability has not been confirmed. In addition, the feasibility of commercial citrus biorefineries has not been validated because the modern biorefinery concept and public acceptance of high-value-added products have not materialized [76,86].

Currently, an important concern in CW biorefining is the creation of a sequentially integrated biorefinery system; the creation of this system depends on the resolution of various problems arising from CW characteristics. For example, the essential oils from citrus fruit waste must be collected and recycled after juice removal, prior to the production of ethanol and methanol. Additionally, before the cellulose, lignin, and hemicellulose from CW can be used for green energy production, high-value products such as pectin, enzymes, single-cell proteins, and organic compounds must be extracted.

Integrated biorefining for sustainable CW management is regarded as a cost-effective and progressive waste management approach that involves conversion to multiple bioactives during the biorefining process [91,92]. Thus, the implementation of CW biorefining is a win-win strategy that offers an integrated approach for giving new value to transformed diverse CW biomass components through environmentally friendly and economically viable tactics, including the valorization of potential waste through a circular bioeconomy [2,93].

## 5. Perspectives and Conclusions

**Figure 3:**
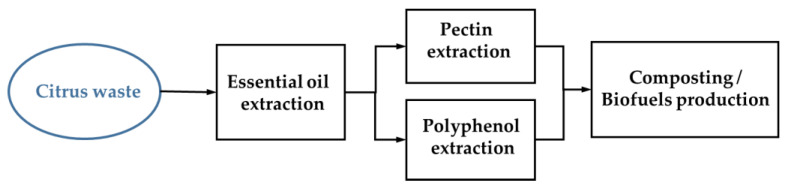
Possible integrated CW biorefinery in Jeju, Korea.

CW management is a major problem in the citrus industry due to environmental pollution resulting from inadequate management of citrus-based waste through existing practices [94,95]. For better sustainability, efforts are needed to change the conventional attitude towards CW, which regards CW as waste that needs to be disposed of rather than as waste containing potentially useful by-products [8,9]. Considering the numerous applications of CW by-products in various sectors, the negative impact of the citrus processing industry on the environment can be reduced if the potential of CW valorization is realized through green economy initiatives. These CWs, which are a rich source of high-value compounds, can be recovered or recycled into high-value products through various valorization strategies for use in food, cosmetics, pharmaceuticals, and environmental management fields. Various environmentally friendly approaches are being developed for effective valorization of CW for extraction of pectin, dietary fiber, polyphenols and essential oils using green technologies and conversion into various high value-added products through biotechnological routes. In this study, we proposed a biorefinery that can directly utilize the biorefinery of CW produced in a limited area of Jeju Island, Korea, for agricultural purposes, produce high value-added products such as EOs, pectin, and polyphenol, and produce bioenergy. Biological conversion of CW to biofuel is the most promising evaluation approach, but its application is mainly limited to laboratory scale. Therefore, efforts are needed to expand the conversion process to an industrial scale through in-depth analysis of the process. An appropriate assessment of environmental sustainability issues related to CW management is necessary, particularly with regard to life cycle assessment [8,96]. The citrus processing industry will spend a significant portion of its current budget for processing residues of CW. These costs can be minimized by adopting sustainable solutions combined with the most appropriate technologies for processing and value addition, depending on location-specific conditions [12]. Taking into consideration the various factors outlined above, the initial proposal for an integrated biorefinery system utilizing CW in Jeju, Korea could be structured as follows: Firstly, the extraction of EOs from CW would be prioritized due to D-limonenine, a constraint in composting or biofuel production. Optionally, the production of pectin or polyphenols could be undertaken, subject to technical and economic considerations. Finally, the process of producing compost or biofuels could utilize the by-product from which D-limonenine has been removed (Figure 3). Additionally, more research is needed to establish cost-effective and innovative integrated biorefinery approaches for full valorization of CW at industrial scale. Regarding resource utilization, despite numerous obstacles already being recognized and addressed, significant work is still needed to commercialize these valorization strategies that can contribute to a sustainable world.

## Figures and Tables

**Table 1:** Content of selected metabolites in mandarin.

Metabolites	Content
Sugars (mg/g FW)	110–140
Glucose (mg/g FW)	10–40
Fructose (mg/g FW)	10–30
Sucrose (mg/g FW)	60–80
Citric acid (g/L)	8–12
Malic acid (g/L)	0.8–2
Vitamin C (mg/g)	0.20–0.57
Carotenoids (μg/g FW)	12–40
Flavonoids (mg/g FW)	0.20–1.0
Phenolic acids (mg/g FW)	0.50−0.85

Data from Lado et al. [19].

**Table 2:** Production and processed citrus in Korea (tons).

**Year**	**Mandarin**	**Tangor ^1^**	**Sum**
2021	520,135 (65,240)	92,983 (1040)	613,118 (66,280)
2020	572,486 (76,989)	82,378 (663)	654,864 (77,602)

^1^ Tangor (*C. reticulata* × *C. sinensis*) is a citrus fruit hybrid of mandarin orange (*Citrus reticulata*) and sweet orange (*Citrus sinensis*). The parenthesis indicates waste.

## Data Availability

Not applicable.
